# Evidence for single-dose protection by the bivalent HPV vaccine—Review of the Costa Rica HPV vaccine trial and future research studies

**DOI:** 10.1016/j.vaccine.2017.12.078

**Published:** 2018-08-06

**Authors:** Aimée R. Kreimer, Rolando Herrero, Joshua N. Sampson, Carolina Porras, Douglas R. Lowy, John T. Schiller, Mark Schiffman, Ana Cecilia Rodriguez, Stephen Chanock, Silvia Jimenez, John Schussler, Mitchell H. Gail, Mahboobeh Safaeian, Troy J. Kemp, Bernal Cortes, Ligia A. Pinto, Allan Hildesheim, Paula Gonzalez, for the Costa Rica HPV Vaccine Trial (CVT) Group

**Affiliations:** a National Cancer Institute, NIH, Bethesda, MD, USA; b Prevention and Implementation Group, International Agency for Research on Cancer, Lyon, France; c Agencia Costarricense de Investigaciones Biomédicas (ACIB), formerly Proyecto Epidemiológico Guanacaste, Fundación INCIENSA, San José, Costa Rica; d Independent Consultant, San José, Costa Rica; e Information Management Systems, Silver Spring, MD, USA; f Roche Molecular Diagnostics, Pleasanton, CA, USA; g HPV Immunology Laboratory, Leidos Biomedical Research, Inc., Frederick National Laboratory for Cancer Research, Frederick, MD, USA

**Keywords:** Human papillomavirus, HPV, Vaccine, Prevention, Cervical cancer, HPV-driven cancers, Reduced dose

## Abstract

The Costa Rica Vaccine Trial (CVT), a phase III randomized clinical trial, provided the initial data that one dose of the HPV vaccine could provide durable protection against HPV infection. Although the study design was to administer all participants three doses of HPV or control vaccine, 20% of women did not receive the three-dose regimens, mostly due to involuntary reasons unrelated to vaccination. In 2011, we reported that a single dose of the bivalent HPV vaccine could be as efficacious as three doses of the vaccine using the endpoint of persistent HPV infection accumulated over the first four years of the trial; findings independently confirmed in the GSK-sponsored PATRICIA trial. Antibody levels after one dose, although lower than levels elicited by three doses, were 9-times higher than levels elicited by natural infection. Importantly, levels remained essentially constant over at least seven years, suggesting that the observed protection provided by a single dose might be durable. Much work has been done to assure these non-randomized findings are valid. Yet, the group of recipients who received one dose of the bivalent HPV vaccine in the CVT and PATRICIA trials was small and not randomly selected nor blinded to the number of doses received. The next phase of research is to conduct a formal randomized, controlled trial to evaluate the protection afforded by a single dose of HPV vaccine. Complementary studies are in progress to bridge our findings to other populations, and to further document the long-term durability of antibody response following a single dose.

## 1 Introduction

Cervical cancer affects more than half a million women annually, with 88% of mortality occurring in low-income nations, where cervical cancer is the third leading cause of cancer mortality in women [1]. If current trends go unabated, the absolute number of cases is expected to increase due to population growth and aging [2], yet, tools to interrupt this trajectory are available. The 70th World Health Assembly endorsed an updated list of evidence-based interventions to be used in the prevention and control of some of the world's deadliest diseases, including cancer [3]. Vaccinating girls aged 9–13 years against human papillomavirus (HPV) and screening women aged 30–49 years for cervical cancer were named as some of the most cost-effective and feasible for implementation [3].

HPV vaccines were licensed and recommended a decade ago [4], in order to reduce individual- and population-prevalence of HPV, a necessary cause of cervical carcinogenesis [5]. These vaccines were initially tested and approved in three-dose regimens [4]. Vaccine uptake has been poor in many world regions [6], likely the consequence of high costs and the intensive infrastructure required for administering three doses over a six-month period. In time, serological data provided consistent evidence that two doses administered among adolescents (9–14 year olds) at least six-months apart evoked immunological responses that were non-inferior compared to three doses among the 16-to 26-year-old women who experienced protection in the trials [7], [8]. Consequently, the European recommending bodies reduced the dosing recommendation for adolescents to two doses in 2014 [9]; the US made a parallel recommendation in 2016 [10].

The Costa Rica Vaccine Trial (CVT) [11] and the PATRICIA Trial [12], both of which tested the bivalent HPV vaccine, showed similar vaccine efficacy for four years in *post hoc* analyses, even among women who received a single dose of the HPV vaccine. Further, in the CVT stable antibody responses were documented at levels between five- and ninefold higher for HPV 16 and 18 than those induced by natural immunity; yet, they were fourfold lower compared with levels elicited by three doses [13]. We have now extended our evaluation of reduced-dose HPV vaccine protection and immunogenicity to seven years in order to document durability of protection [14], an important determinant of the long-term impact of a vaccination program.

At present, HPV vaccine uptake and cervical cancer screening implementation has been insufficient in most world regions and the expected number of cervical cancers is projected to increase over the coming decades [6]. We hypothesize that one-dose HPV vaccination, if sufficiently efficacious, would make broader vaccination of the neediest populations a reality.

The objective of this manuscript is to summarize the evidence to date for single-dose efficacy of the bivalent HPV vaccine from *post hoc* analysis of the CVT, review the validity of these findings by discussing potential biases, and present our future efforts to additionally address critical questions around single-dose protection afforded by the HPV vaccines.

## 2 Methods

### 2.1 Study population

CVT was a publicly funded, four-year, community-based, randomized phase III clinical trial (registered with Clinicaltrials.gov NCT00128661) [15]. From 2004 to 2005, 7466 women were consented and randomized to receive either the AS04-HPV-16/18 vaccine (Cervarix®, GlaxoSmithKline Biologicals, Rixensart, Belgium) or a control hepatitis A vaccine (Havrix®,GlaxoSmithKline Biologicals) in a 1:1 ratio at 0, 1, and 6 months. Participants were followed annually for 4 years, with more frequent follow-up when clinically indicated. Protocols were approved by the Institutional Review Boards (IRB) of the U.S. National Cancer Institute, the Costa Rican INCIENSA (for the CVT) and the National University Review Board (for the Long-term follow-up [LTFU] component), and all participants signed informed consent.

### 2.2 Study design

At enrollment and follow-up visits, participants provided a serum sample, and for sexually-experienced women, a pelvic exam was performed at which time cervical cells were collected for cytology and HPV DNA testing. At the end of the four-year trial, participants were offered the vaccine they had not received at enrollment (cross-over vaccination) and were invited to stay in a long-term follow-up observational study [16]. During this observational study, HPV-vaccinated participants were followed biennially for six additional years, where each clinic visit consisted of a pelvic exam with collection of a cervical sample, and a serum sample. To replace the original control group, this observational study recruited 2836 unvaccinated women from the same birth cohorts and geographic regions as the original trial participants into an Unvaccinated Control Group (UCG) who were also followed biennially. We extensively documented that this new control group had similar characteristics to the trial participants, particularly with regard to risk of HPV acquisition [9].

As part of the study design, time windows for each vaccine dose were pre-defined based on the first vaccination date. Women who became pregnant during the vaccination phase or who were referred to colposcopy were deferred, and missed that dose if the vaccination window was closed; this occurred in roughly 20% of women in the CVT [11]. Reasons for missing vaccine doses are discussed in the results section of this manuscript.

In this report, we summarize the published data to date. We compared multiple vaccine groups with their corresponding control groups, as follows: (i) women who received one HPV16/18 vaccine dose; (ii) women who received two HPV16/18 vaccine doses at enrollment and 1 month later; (iii) women who received two HPV16/18 vaccine doses at enrollment and 6 months later; (iv) women who received all three HPV16/18 vaccine doses; (v) women randomized to the original control arm; and (vi) women from the new unvaccinated control group. We evaluated these groups for virologic and serologic endpoints.

### 2.3 Laboratory methods

HPV DNA detection and genotyping from cervical specimens were performed at DDL Diagnostic Laboratory [17], [18], [19]. Extracted DNA was used for PCR amplification with the SPF10 primer sets. The same SPF10 amplimers were used on SPF10-DEIA–positive samples to identify HPV genotype by reverse hybridization on a line probe assay (LiPA; SPF10-DEIA/HPVLiPA25, version 1; Labo Bio-Medical Products, Rijswijk, the Netherlands), which detects 25 HPV genotypes.

HPV16 and HPV18 serum antibody levels were measured by ELISA using HPV16 and HPV18 virus like particle (VLP) at the NCI HPV Immunology Laboratory, as previously described [13]. The laboratory-determined seropositivity cut-offs for HPV16 and HPV18 were 8 EU/mL and 7 EU/mL, respectively. Laboratory-blinded replicates were included in each batch and the inter-plate coefficient of variation (CV) was ≤10%.

HPV16 avidity was measured in serum by coating plates with HPV16 L1 VLP. Each serum sample was tested at a dilution that yielded an absorbance reading of 1.0 ± 0.5 as previously determined in an HPV16 VLP ELISA. Guanidine-HCl (GuHCl) was added to the samples at various concentrations (0.5–3.5 M); the concentration of GuHCl that reduced the optical density by 50%, compared with sample wells without GuHCl treatment, defined the Avidity Index.

HPV16 and HPV31 neutralization titers were determined using a previously described pseudovirion-based secreted alkaline phosphatase neutralization (SEAP) assay [13], using specimens collected at the last (48 month) clinic visit.

### 2.4 Statistical analysis

For analyses of the efficacy of <3 doses during the randomized, blinded phase (first four years of study), the primary endpoint was newly detected HPV 16 or 18 infection that persisted for at least 6 months. Endpoint definition required detections of the same genotype consecutively at least four months apart with no intervening negatives. We required detection of the first infection to start at the 12-month study visit or later to avoid prevalent infections at enrollment and differential assessment by missed visits during the vaccination phase (i.e.: possible bias from assessing outcomes differentially for women who missed or received the six-month vaccination). We additionally investigated 12-month persistent HPV16/18 infections, and HPV 31/33/45 infections (after excluding women with prevalent HPV31, 33 and 45 infections detected at enrollment), HPV types with prior evidence of vaccine cross-protection.

The analytic cohort excluded women who were both HPV16 and 18 DNA positive at enrollment, and women with no follow-up visits post-enrollment (for analyses of cross- protection the analytic cohort was restricted to women who were HPV DNA negative for types 31, 33, or 45 at enrollment instead of restricting to those who were DNA negative for HPV16 or 18 at enrollment). Within each dose group, the complement of the ratios of the attack rates for the HPV arm and the control arm are the vaccine efficacy (VE) estimates. Instead of conducting a direct comparison by number of doses within the HPV arm only, data from the randomized control arm were used, because we were uncertain whether the underlying HPV attack rates would vary by vaccine dose (i.e.: are women who missed dose(s) riskier in some way, and therefore have a higher HPV attack rate?).

For analyses occurring at the seven-year study visit, multiple endpoints were assessed, including year-7 incident and prevalent HPV infections. This focus on the year seven results and not cumulative assessment of the endpoints over the seven years of the study follow-up was meant to ensure the assessment of the longevity of the protection against HPV infections, instead of allowing the early-term protection to potentially drive the longer-term findings. Comparisons of endpoints are made between the HPV dose groups and the new, non-randomized unvaccinated control group, since the original control group was exited by this time point. For each of the endpoints, we reported the number (n) of women with the endpoint, the total number (N) of eligible women, and corresponding percentage (%) by each of the four HPV vaccine groups. We also report the p-values comparing rates in the 2-dose (0/6 month), 2-dose (0/1 months), and 1-dose groups with the rate in the 3-dose group using Fisher’s test. Given the use of the non-randomized control group, we present a comparison of HPV prevalence by group in lieu of VE.

## 3 Results

### 3.1 Ruling out bias and confounding

Data generated from CVT and other sources on single-dose HPV vaccine protection are being used to make decisions about future studies. While dose-specific data were obtained in the context of a randomized clinical trial, they are observational in nature. A concern is that one-dose protection is not actually a function of the HPV vaccine, but instead related to an underlying characteristic shared by women who received only one dose. We provide the following example: perhaps women who received a single dose did so because they had a strong adverse reaction to the vaccine, but are actually better able to mount an immune response to the first dose. If this were the case, then our findings on single-dose protection would not generalize to the majority of women who can tolerate multiple HPV vaccine doses.

To confirm the validity of the findings to date, several metrics have been used to evaluate potential biases and confounding in our data (Table 1), including by dose assessment of:

• Demographic and HPV-related differences at enrollment, including sexual behavior and presence or absence of Chlamydia trachomatis by dose group;
• Reasons for missed doses;
• Vaccine antibody response elicited one month after the first dose, when all women received the same number of doses irrespective of the total number of doses they received; and,
• Prevalence of HPV genotypes not protected by the vaccine, as an indicator of HPV exposure, accumulated over the four-years of follow-up.

**Table 1:** Threats to validity of single-dose HPV protection, and evaluations of bias and confounding within these rubrics.

Threat to validity	Evaluation of bias and confounding
Are women who received a single-dose of the HPV vaccine different from women who received a single-dose of the control vaccine?	Within the one-dose arm, women who were in the HPV and control arms were similar with regard to age, number of clinic visits, HPV16/18 DNA- and sero-status, and prevalence of Chlamydia trachomatis

Did single-dose women receive less than a complete schedule for reasons related to HPV vaccination?	Assessment of reasons for missed doses revealed that most reasons were involuntary and unrelated to randomization arm, such as pregnancy and colposcopy referral. It was less common for participants to refuse the vaccine or have a medical condition that was contraindicated to vaccination

Are women who received a single-dose of the HPV vaccine immunologically different from women who received multiple doses of the HPV vaccine?	Compared to the two and three-dose groups, women in the one-dose HPV group had similar HPV antibody titers following the initial HPV vaccine dose, when all women received the same number of doses

Is HPV exposure during the follow-up phase similar among women who received a single-dose of the HPV vaccine compared to the control HPV vaccine or other dose groups?	Cumulatively over the first four years of follow-up, women in the active control arm had the same HPV attack rate regardless of the number of doses received. Seven years after initial vaccination, women in the HPV arm had similar prevalence of non-vaccine HPV genotypes, a metric of HPV exposure, independent of dose group

#### 3.1.1 Enrollment characteristics

Based on the enrollment characteristics, we evaluated whether balance was present by vaccine received (i.e.: comparing participants randomized to the HPV arm versus the control arm) within a dose group. There were no differences by arm in the one dose group by age at vaccination (p = 0.9), number of clinic visits attended (a metric for increased opportunities for endpoint assessment; p = 0.9), or HPV16/18 DNA (p = 0.8) or serologic status (p = 0.5). We also did analyses to investigate sexual risk-taking behavior using presence or absence of Chlamydia trachomatis (Ct)- the prevalence of Ct was balanced within a dose group by arm (p = 0.8; Table 2).

**Table 2:** Balance in enrollment characteristics by vaccine arm and number of vaccine doses received.

	One dose			Two doses (0/1)			Two doses (0/6)			Three doses		
Group (N)	HPV (277)	Control (274)	p	HPV (382)	Control (364)	p	HPV (106)	Control (77)	p	HPV (2965)	Control (3021)	p
*Age*												
≤20 or less	163 (58.8%)	163 (59.5%)	0.9	233 (61.0%)	224 (61.5%)	0.9	64 (60.4%)	45 (58.4%)	0.8	1679 (56.6%)	1718 (56.9%)	0.9
≥21 or older	114 (41.2%)	111 (40.5%)		149 (39.0%)	140 (38.5%)		42 (39.6%)	32 (41.6%)		1286 (43.4%)	1303 (43.1%)	
p										*0.3*	**0.3**	

*Non-vaccine followup study visits*												
0	87 (31.4%)	94 (34.3%)	0.9	65 (17.0%)	60 (16.5%)	0.8	4 (3.8%)	4 (5.2%)	0.03	49 (1.7%)	38 (1.3%)	0.3
1–3	46 (16.6%)	42 (15.3%)		66 (17.3%)	60 (16.5%)		15 (14.2%)	18 (23.4%)		366 (12.3%)	401 (13.3%)	
4	44 (15.9%)	47 (17.2%)		79 (20.7%)	66 (18.1%)		23 (21.7%)	14 (18.2%)		640 (21.6%)	615 (20.4%)	
5	68 (24.5%)	63 (23.0%)		117 (30.6%)	126 (34.6%)		50 (47.2%)	21 (27.3%)		1354 (45.7%)	1361 (45.1%)	
6+	32 (11.6%)	28 (10.2%)		55 (14.4%)	52 (14.3%)		14 (13.2%)	20 (26.0%)		556 (18.8%)	606 (20.1%)	
p										*<0.0001*	**<0.0001**	

*HPV16/18 DNA positivity*												
Negative	244 (88.4%)	238 (87.8%)	0.8	339 (88.7%)	332 (91.5%)	0.2	99 (93.4%)	70 (90.9%)	0.5	2737 (92.5%)	2749 (91.1%)	0.05
Positive	32 (11.6%)	33 (12.2%)		43 (11.3%)	31 (8.5%)		7 (6.6%)	7 (9.1%)		223 (7.5%)	270 (8.9%)	
p										*0.01*	**0.3**	

*HPV16/18 seropositivity*												
Negative	166 (61.5%)	154 (58.6%)	0.5	222 (59.5%)	214 (60.3%)	0.8	60 (58.3%)	43 (58.1%)	1.0	1834 (63.2%)	1829 (62.0%)	0.3
Positive	104 (38.5%)	109 (41.4%)		151 (40.5%)	141 (39.7%)		43 (41.7%)	31 (41.9%)		1066 (36.8%)	1122 (38.0%)	
p										*0.4*	**0.6**	

*Chlamydia trachomatis*												
Negative	245 (91.4%)	242 (90.6%)	0.8	321 (85.4%)	318 (88.1%)	0.3	92 (86.8%)	64 (83.1%)	0.5	2646 (89.7%)	2657 (88.3%)	0.09
Positive	23 (8.6%)	25 (9.4%)		**55** (14.6%)	43 (11.9%)		14 (13.2%)	13 (16.9%)		303 (10.3%)	351 (11.7%)	
p										*0.04*	**0.3**	

*Lifetime # of sex partners*												
0–1	149 (53.8%)	131 (48.3%)	0.4	165 (43.3%)	187 (51.8%)	0.03	62 (58.5%)	42 (54.5%)	0.9	1631 (55.0%)	1702 (56.5%)	0.5
2	59 (21.3%)	63 (23.2%)		91 (23.9%)	85 (23.5%)		17 (16.0%)	14 (18.2%)		611 (20.6%)	591 (19.6%)	
3+	69 (24.9%)	77 (28.4%)		125 (32.8%)	89 (24.7%)		27 (25.5%)	21 (27.3%)		721 (24.3%)	722 (23.9%)	
p										*0.001*	**0.1**	

*Lifetime # of pregnancies*												
0	152 (54.9%)	149 (54.4%)	0.7	197 (51.6%)	202 (55.5%)	0.4	55 (51.9%)	48 (62.3%)	0.4	1541 (52.0%)	1563 (51.7%)	0.3
1	67 (24.2%)	74 (27.0%)		112 (29.3%)	106 (29.1%)		32 (30.2%)	19 (24.7%)		861 (29.0%)	922 (30.5%)	
2+	58 (20.9%)	51 (18.6%)		73 (19.1%)	56 (15.4%)		19 (17.9%)	10 (13.0%)		563 (19.0%)	536 (17.7%)	
p										*0.8*	**0.4**	

*Oral contraceptive use*												
Never	117 (42.2%)	99 (36.7%)	0.2	139 (36.4%)	136 (37.5%)	0.8	49 (46.2%)	32 (41.6%)	0.5	1169 (39.5%)	1202 (39.9%)	0.8
Yes	160 (57.8%)	171 (63.3%)		243 (63.6%)	227 (62.5%)		57 (53.8%)	45 (58.4%)		1789 (60.5%)	1809 (60.1%)	
p										*0.2*	**0.6**	

*Smoking*												
Never	246 (88.8%)	232 (85.0%)	0.4	303 (79.3%)	305 (83.8%)	0.1	93 (87.7%)	65 (84.4%)	0.1	2569 (86.7%)	2628 (87.2%)	0.5
Former	15 (5.4%)	**20** (7.3%)		34 (8.9%)	19 (5.2%)		7 (6.6%)	2 (2.6%)		160 (5.4%)	170 (5.6%)	
Current	16 (5.8%)	21 (7.7%)		45 (11.8%)	40 (11.0%)		6 (5.7%)	10 (13.0%)		235 (7.9%)	217 (7.2%)	
p										*0.004*	**0.06**	

Three sets of p values are provided: one is a test for differences by arm within dose, one is a test across dose in the HPV arm (in italics), and one is a test across dose in the HAV arm (in bold). The p-values in the separate columns are for the HPV arm vs Control arm comparisons within a dose group and p-values in the 3-dose column are for the across dose group comparisons within an arm.

Some differences are noted across HPV-vaccinated groups, with single-dose recipients having more HPV16/18 DNA positivity at enrollment compared to three-dose HPV vaccine recipients (p = 0.01 for the HPV arm); HPV16/18 seropositivity was not different across the dose groups.

#### 3.1.2 Reasons for receiving fewer doses

Among all vaccinated women, reasons for not receiving all doses were similar in both HPV and HAV arms conditional on the number of doses received (Table 3, ref 11). The most common reasons for not receiving all three doses were involuntary, including pregnancy and colposcopy referral (∼35% of instances); it was less common for participants to refuse the vaccine.

**Table 3:** Reasons for missed dosing at one month and six months, among women who received one of two doses of the vaccine, by arm.

	Missed dose at 1 month		Missed dose at 6 months	
	HPV arm N (%)	HAV arm N (%)	HPV arm N (%)	HAV arm N (%)
Pregnancy	35 (9.1)	35 (10.0)	205 (31.1)	202 (31.7)
Colposcopy referral	58 (15.1)	46 (13.1)	69 (10.5)	53 (8.3)
Medical condition	61 (15.9)	67 (19.1)	110 (16.7)	116 (18.2)
Vaccine refusal	42 (11.0)	38 (10.8)	150 (22.8)	142 (22.3)
Missed Visit	122 (31.9)	98 (27.9)	54 (8.2)	76 (11.9)
Other	65 (17.0)	67 (19.1)	71 (10.8)	49 (7.7)

The three most common ‘other’ reasons included: woman could not get time off work, personal reasons, woman not using an acceptable form of birth control.

#### 3.1.3 Antibody levels when all dose groups received only one dose

The antibody levels measured at one-month following the initial doses, when all women received the same number of doses irrespective of the total number of doses they received, were not significantly different (13). Specifically, the HPV16 GMTs were 419.7 (95% CI 251.0 to 701.7) for 1 dose, 646.2 (95% CI 478.2 to 873.4) for 2 doses, and 597.0 (95% CI 454.1 to 784.8) for 3 doses (p = .4); the respective data for HPV18 were 207.0 (95% CI 114.9 to 372.8), 244.1 (95% CI 184.2 to 323.4), and 207.9 (95% CI 163.3 to 264.5) (p = .7). This allayed concerns that the one-dose recipients may have had a more robust intrinsic ability to respond to the vaccine.

#### 3.1.4 HPV infections during the follow-up

After four years of follow-up, in the HAV arm, the attack rates of incident HPV16 or HPV18 infections that persisted for at least six months were similar among women who received three doses (7.6%; 95% CI: 6.7–8.6%), two doses (6.3%; 95% CI: 4.2–9.1%), or one dose (8.0%; 95% CI: 4.7–12.5%) indicating that they were at similar risk for acquiring HPV infections regardless of the number of HAV doses they received [11]. Since balance in enrollment characteristics (Table 2) was observed between the HPV and HAV arms indicating successful randomization, we use the transitive property to infer that there was likely balance in HPV 16/18 exposure by dose group among the HPV-vaccinated arm. Further, assessment of HPV genotypes not protected by the bivalent HPV vaccine showed balance at both years 4 and 7, indicating continued equality in HPV exposure [11], [14]. In the four-year analysis [11], the cumulative detection of carcinogenic HPV types excluding HPV16/18/31/33/45 was 14.9% (95% CI: 13.6–16.2%) for women who received three doses, 14.1% (95% CI: 11.0–17.6%) for women who received two doses, and 12.7% (95% CI: 8.6–17.9%) or women who received one dose. At year seven [7], the point prevalence for the same group of HPV types was 15.2% (95% CI: 13.7–16.8%) for women who received three doses, 14.3% (95% CI: 10.5–18.9%) for women who received two doses (at 0/6), and 13.4% (95% CI: 8.4–20.0%) for women who received one dose [14].

### 3.2 Evidence of protection against virologic outcomes

In evaluations of single-dose efficacy using the bivalent HPV vaccine, the data were assessed at two timepoints: first, during the initial four-year randomized blinded phase that included the randomized control arm (although not randomized by dose) to assess background rates of HPV infection, and then at seven years in our long-term follow up study that included a new observational control arm. The analytic strategy changed between the two timepoints. At four years, we cumulatively assessed HPV infections over the four-year follow up. At the seven-year data point, we assessed point prevalence as the outcome. The goal was to assess continued duration of protection; so, it was important to avoid having protection documented in the initial four years’ drive findings in the latter years. In future analyses, we will continue to use this approach of assessing protection at the far-out time point.

**Fig. 1:**
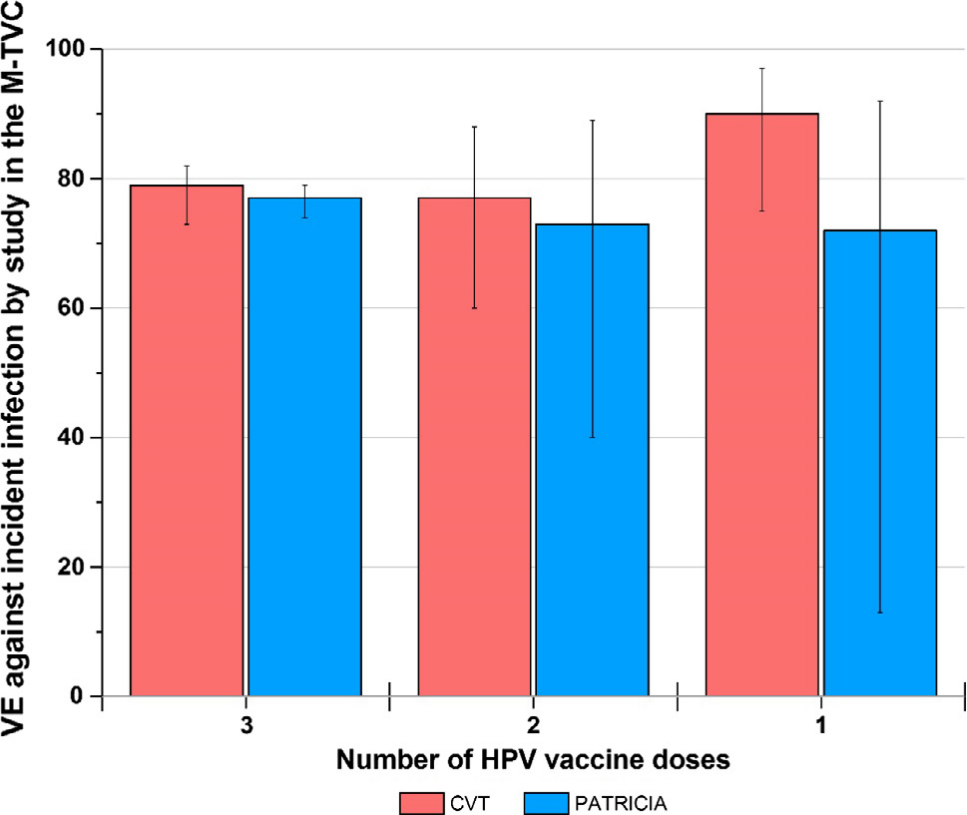
Four-year efficacy against incident HPV16/18 infections, by dose group, in the CVT and PATRICIA trials. Legend. The endpoint assessed was cumulative HPV16 or 18 infections in an analytical cohort of women who were HPV16 and 18 DNA negative at the enrollment visit. VE Vaccine efficacy M-TVC Modified total vaccinated cohort.

Four years after initial vaccination, a single dose of the bivalent HPV vaccine had comparable efficacy to three doses of the vaccine using an endpoint of cumulative persistent HPV infection (11). The four-year efficacy against HPV16 or 18 infections (that persisted for at least six months) among women who were HPV DNA negative for these types at first vaccination: for three doses = 84% (95% CI = 77–89%; 37 and 229 events in the HPV [n = 2957] and control [n = 3010] arms, respectively); two doses = 81% (95% CI: 53–94%); 5 and 24 events among HPV [n = 422] and control [n = 380] arms, respectively); and one dose = 100% (95% CI: 79–100%; 0 and 15 events among HPV [n = 196] and control [n = 188] arms, respectively. These findings were independently confirmed using data from the PATRICIA trial, where women who received one dose had the same VE as two and three doses [12]. It should be noted that in comparisons of VE by dose group, we benchmark the one-dose VE against that of three-doses (the historical gold standard) instead of interpreting the absolute VE, which is influenced by the cohort and endpoint chosen for the analysis. In Fig. 1, four-year efficacy against an endpoint of cumulative incident HPV16/18 infection (n.b.: the endpoint is not persistent infection, which is why the point estimate decreases) hovers around 80% for all dose groups—this does not mean that is the anticipated level of protection for one-dose HPV vaccination, but instead, demonstrates that one-dose HPV VE is not inferior to three-dose VE among the same analytic population and utilizing the same endpoint for analyses.

**Fig. 2:**
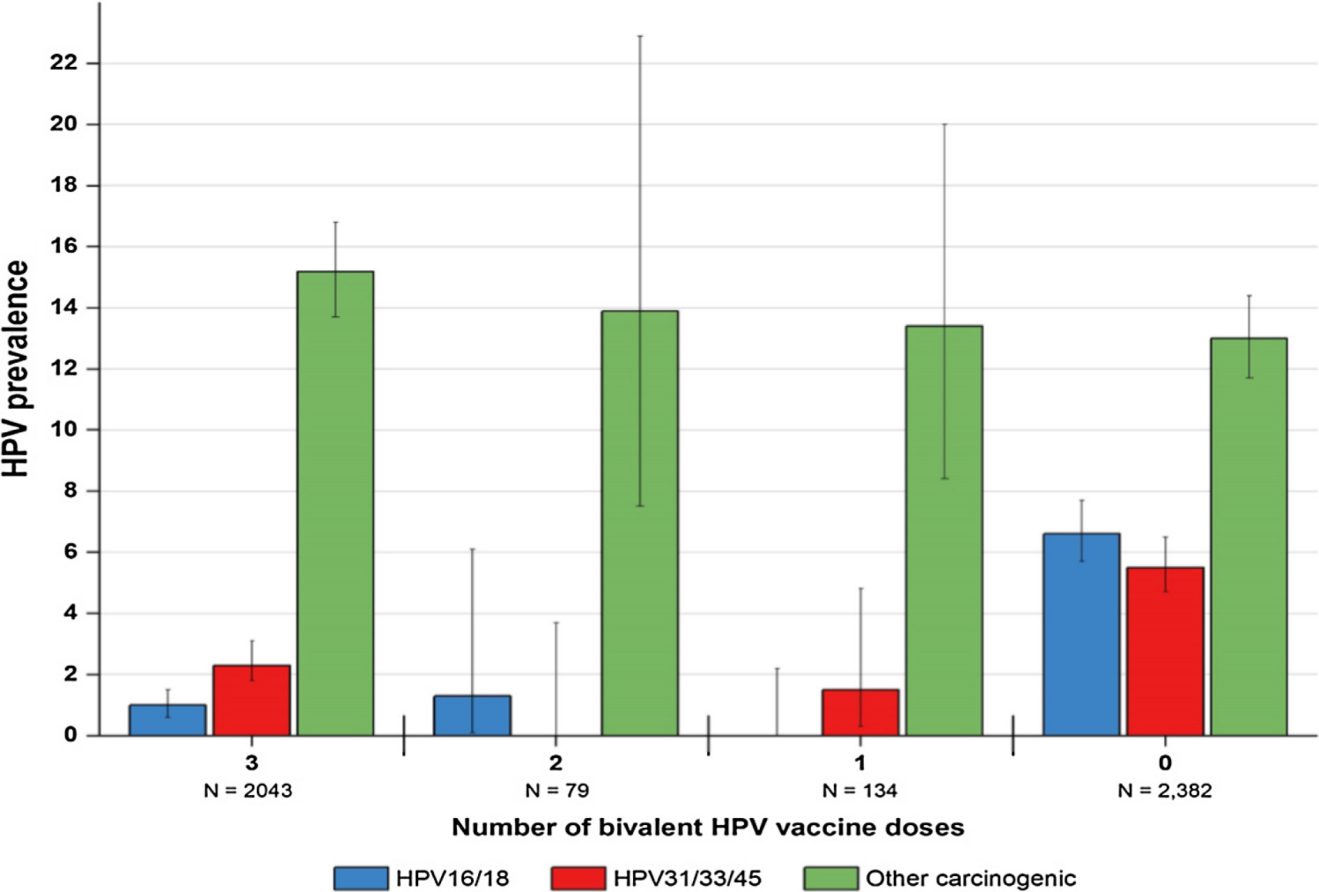
HPV prevalence measured seven years after initial vaccination among women who received 3, 2, 1, and 0- doses in the Costa Rica HPV Vaccine Trial. Legend. The endpoint was HPV16 or 18 infections detected seven years following enrollment among the HPV vaccine groups and the contemporaneous visit among the unvaccinated control group. This was assessed among the total vaccinated cohort and the unvaccinated control group.

We now have data from women in the CVT out to seven years after their initial vaccination [14]. The most recent results, from that 7th year, show that the initially strong protection observed by a single dose of the bivalent HPV vaccine indicates no evidence of diminishing. Single-timepoint infection rates by types targeted by the vaccine remain remarkably low. Among the participants who received a single dose, there were zero HPV 16/18 cervical infections detectable at year 7 (Fig. 2). This is similar in women who received the three-dose regimen, where there were 20 (1.0%) HPV 16/18 infections. For comparison, the HPV prevalence among the unvaccinated women was considerably higher for HPV 16/18 (6.6%), suggesting that even a single dose is continuing to provide robust protection. Again, carcinogenic HPV types not protected against by the HPV vaccine were detected with similar frequency among vaccinated (15.0%) and unvaccinated (13.0%) women, indicating similar exposure to HPV infections.

Our assessments of protection afforded by fewer doses also included cross protection against vaccine-related types HPV 31/33/45. In our initial assessment in a pooled analysis of the CVT and PATRICIA trials, after four-years of follow-up [12], cross-protective efficacy was assessed among all women after excluding those who were HPV DNA-positive for HPV-31/33/45 infections at the enrolment visit. Vaccine efficacy against one-time detection of incident HPV-31/33/45 infections was 59.7% (95% CI: 56.0–63.0%) for three doses, 37.7% (12.4–55.9%) for two doses, and 36.6% (−5.4 to 62.2%) for one dose. We further classified by timing of the second vaccine dose and observed no vaccine efficacy for women who received their second dose 1 month after dose one, whereas women who received their second dose 6 months after dose one had a higher efficacy estimate. Based on these findings, we noted that cross-protective efficacy might require two doses administered at least six months apart, and might be lost with a single-dose HPV vaccine administration. Then, in the analysis of CVT data seven years following initial HPV vaccination, the prevalence of HPV31/33/45 were similar between 3-dose (2.3%; 95% CI: 1.8–3.1%; referent group), 2-dose (0/6 months; 0.0%; 95%CI: 0.0–3.7%; p = .26 compared to three doses) and 1-dose groups (1.5%; 95% CI: 0.3–4.8%; p = .77); these were against a background prevalence in the control group of 5.5% (95% CI: 4.7–6.5%). We have considered these differences in the interpretation of the cross-protective efficacy data at four and seven years and noted that, in the four-year data, there were imbalances in HPV31/33/45 prevalence in the controls groups for the different dosing schedules that may have confounded the efficacy assessments. At present, further investigation is warranted on efficacy of cross-protection for a single dose. Yet, regardless of what is ultimately determined, it is important to remember that protection against the HPV16/18 in fewer dose schedules would provide a clear benefit, given that these two HPV types account for the vast majority of all cervical cancers worldwide.

### 3.3 Serum antibody patterns

**Fig. 3:**
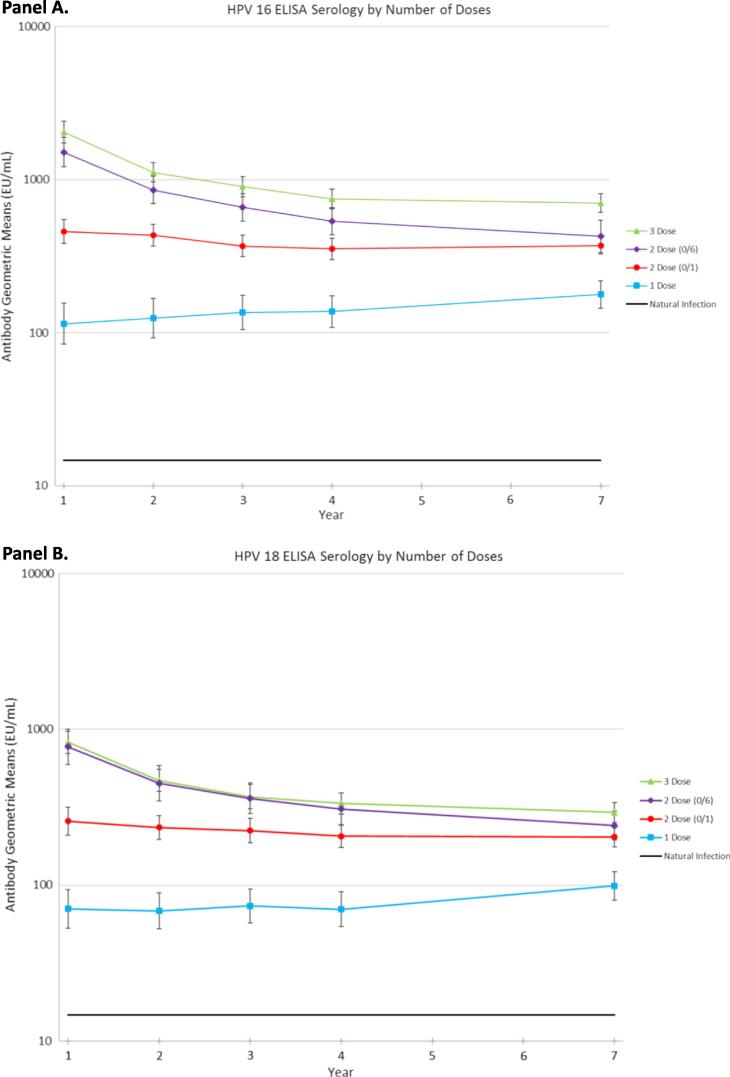
Human Papillomavirus (HPV) type 16 (panel A) and type 18 (panel B) antibody levels up to seven years following initial HPV vaccination, by number of doses received.

Among women who received a single dose, 100% seroconverted, and HPV16 and HPV18 antibody titers (assessed by ELISA) were substantially higher than those among naturally-infected unvaccinated women (approximately ninefold higher for HPV16 and fivefold higher for HPV18) four years after initial vaccination. Titers remained stably elevated at our latest assessment, seven years post-vaccination, albeit still at four- to fivefold lower levels than for two or three doses [14] and an order of magnitude higher than those elicited by natural HPV infections (Fig. 3).

Neutralizing antibodies, the presumed mediators of protection, were measured via SEAP at year 4. They were highly correlated with levels measured by ELISA: spearman correlations were high for three (0.87), two (0/1; 0.72), two (0/6; 0.80), and 1 (0.79) dose groups, although decreased correlation was noted for the fewer-dose groups [20]. By the SEAP assay, HPV16 seropositivity was greater than 95% for all HPV-dose groups, and was no different by dose group (p = 0.81).

HPV16 VLP antibody avidity, a measure of the quality of the antibody response, was measured at years four and seven. In years four and seven, one dose recipients had 80% of the avidity index compared to those who received three-doses, thus suggesting that HPV16 antibody quality was stable over time [13], [14]). Thus, avidity among one-dose recipients also appears stable over time.

## 4 Discussion – future directions

Since 2011, the CVT and PATRICIA trials have provided evidence that a single dose of the bivalent HPV vaccine provides strong and lasting protection against HPV16 and 18, and suggest there may be the additional benefit of cross-protection against phylogenetically-related HPV types. Much work has been done to rule out bias and confounding by dose group. Most recently, we extended the documentation on the duration of protection against virologic and immunologic endpoints out to seven years. Yet, we acknowledge that the group of women receiving one dose of the bivalent HPV vaccine in the CVT and PATRICIA trials was relatively small, and that they were not randomized to a reduced-dosing schedule. Based on compelling data together with both the fact that they challenge the prevailing dogma that protein-based subunit vaccines require a prime-boost regimen, and the potential public health impact of an effective one-dose strategy, we contend that further studies are warranted.

Thus, the next phase of our research has three complementary component parts, aimed at providing the rigorous, long-term data to drive HPV vaccine recommendations to one dose if warranted. The three parts include (1) extension of the follow-up of the one-dose women from the original Costa Rica HPV Vaccine Trial (“CVT EXTEND”), (2) a new RCT that formally evaluates the protection of a single dose of the HPV vaccines (“ESCUDDO study”; ClinicalTrials.gov identifier: NCT03180034), and (3) immunobridging trials to other populations around the world. Short introductions to these efforts are provided below.

### 4.1 CVT EXTEND

It is critical to continue to evaluate the long-term stability of antibody responses among reduced dose recipients. To do so, we extended the follow-up time of women in the original CVT who received one or two doses and a subset who received three doses of the HPV vaccine out to 15 years (note that women enrolled into CVT were between the ages of 18 and 25). The main aim of this extension is to describe, by dose, the long-term positivity and stability of the antibody response to HPV vaccination. These 15-year data on the durability of the antibody response for a single dose of the HPV vaccine will be available prior to the completion of four-years of follow-up of the ESCUDDO study (described below). Thus, results from both studies will be paired for presentation to recommending bodies.

### 4.2 One-dose HPV vaccine trial, the ESCUDDO study

The US NCI, again in collaboration with the Costa Rica Agencia Costarricense de Investigaciones Biomédicas (formerly Proyecto Epidemiologico Guanacaste), is conducting a large, 20,000 subject, randomized, controlled, non-inferiority efficacy trial in Costa Rica (ClinicalTrials.gov Identifier NCT03180034) of two FDA- and WHO-approved HPV vaccines: the bivalent vaccine Cervarix (GSK) and the nonavalent vaccine Gardasil 9 (Merck). The main goals of the trial are to evaluate whether, in adolescent girls (ages 12 to 16), one dose or two doses of the bivalent or nonavalent vaccines can confer strong, durable protection against persistent HPV infections. Virologic endpoints are necessary in the evaluation of a one-dose schedule, as the antibody levels are inferior to those of two doses, and, as yet, we do not know the minimum level required for protection. Separately for each vaccine, one-dose will be tested for non-inferiority against the two-dose regimen. Analyses will also be conducted to estimate vaccine efficacy versus no vaccination using a concurrent population survey of comparable, unvaccinated age-matched females in the same region, who will be tested for HPV DNA and then immediately vaccinated. The population survey will be used to estimate vaccine efficacy against incident persistent infection by subtracting off estimates of HPV infection prevalence four years earlier. The effort is intended to provide definitive results that can drive widespread recommendations by agencies such as the World Health Organization.

### 4.3 One-dose HPV immunobridging studies

The overall aim of our immunobridging work is to compare dose- and vaccine-induced HPV-specific antibody levels among girls in multiple other countries to the antibody levels observed to provide protection against HPV infection in the Costa Rica ESCUDDO study (described above). These data will address the hypothesis that girls in other countries, such as those in sub-Saharan Africa where more co-morbidities may exist, including parasites, other infections, malnutrition, and general decreased immune status, mount an immune response to the HPV vaccines that is not significantly less than that observed in women in a Western, middle-income country, in this case, Costa Rica. These studies are running in parallel with the ESCUDDO study, to accumulate the necessary data simultaneously.

In addition to the immunobridging potential of the one-dose trial to other world populations, it also creates the needed biobank in which to evaluate/immunobridge to one-dose regimens of future VLP-based biosimilar HPV vaccines.

### 4.4 Summary

From the global perspective, women who are at the greatest lifetime risk of cervical cancer are not being vaccinated. Our data showing that a single dose of the HPV vaccine continued to protect against HPV infection, with documented stability of antibody and avidity up to 7 years, augments other data supporting the hypothesis that one dose may be sufficient. Continued demonstration of the protection afforded by one dose will be provided by the CVT cohort for 15 years post initial HPV vaccination. This durability data is intended to complement the ESCUDDO study, a formal trial of the bivalent and nonavalent HPV vaccines. Finally, the immunobridging studies will focus on regions that may have additional comorbidities to ensure the findings from Costa Rica are generalizable to other world populations. Combined, we intend to provide sufficient evidence to motivate policy change, in the event one-dose HPV vaccination continues to demonstrate robust protection.

From a public health perspective, it is important to plan further studies in light of the current data and the likelihood of the planned trial demonstrating the validity of a single-dose strategy. In this regard, the efficacy of a single-dose strategy could have dramatic public health implications, particularly as it could establish substantial herd immunity and thus protect not only vaccinees but also other against the world’s third leading cause of cancer death in women.

## Funding

The Costa Rica HPV Vaccine Trial is a long-standing collaboration between investigators in Costa Rica and the NCI. The trial is sponsored and funded by the NCI (contract N01-CP-11,005), with funding support from the National Institutes of Health Office of Research on Women's Health. GlaxoSmithKline Biologicals (GSK) provided vaccine and support for aspects of the trial associated with regulatory submission needs of the company under a Clinical Trials Agreement (FDA BB-IND 7920) during the four-year, randomized, blinded phase of our study.

## Notes

The NCI and Costa Rica investigators are responsible for the design and conduct of the study; collection, management, analysis, and interpretation of the data; and preparation of the manuscript. John T. Schiller and Douglas R. Lowy report that they are named inventors on US Government-owned HPV vaccine patents that are licensed to GlaxoSmithKline and Merck and for which the National Cancer Institute receives licensing fees. They are entitled to limited royalties as specified by federal law. The other authors declare that they have no conflicts of interest.

### Investigators in the Costa Rica HPV vaccine trial (CVT) group

Proyecto Epidemiológico Guanacaste, Fundación INCIENSA, San José, Costa Rica—Bernal Cortés (specimen and repository manager), Paula González (LTFU: co-principal investigator), Rolando Herrero (CVT: co-principal investigator), Silvia E. Jiménez (trial coordinator), Carolina Porras (co-investigator), Ana Cecilia Rodríguez (co-investigator).

United States National Cancer Institute, Bethesda, MD, USA—Allan Hildesheim (co-principal investigator & NCI co-project officer), Aimée R. Kreimer (LTFU: co-principal investigator & NCI co-project officer), Douglas R. Lowy (HPV virologist), Mark Schiffman (CVT: medical monitor & NCI co-project officer), John T. Schiller (HPV virologist), Mark Sherman (CVT: QC pathologist), Sholom Wacholder (statistician).

Leidos Biomedical Research, Inc., Frederick National Laboratory for Cancer Research, Frederick, MD, USA (HPV Immunology Laboratory)—Ligia A. Pinto, Troy J. Kemp

Georgetown University, Washington, DC, USA—Mary K. Sidawy (CVT: histopathologist)

DDL Diagnostic Laboratory, Netherlands (HPV DNA Testing)—Wim Quint, Leen-Jan van Doorn, Linda Struijk.

University of California, San Francisco, CA, USA- Joel M. Palefsky (expert on anal HPV infection and disease diagnosis and management), Teresa M. Darragh (pathologist and clinical management)

University of Virginia, Charlottesville, VA, USA- Mark H. Stoler (QC pathologist)
